# Adapt and advance: the Medical Library Association's journey through innovation and change

**DOI:** 10.5195/jmla.2024.2010

**Published:** 2024-07-01

**Authors:** Kevin Baliozian

**Affiliations:** 1 Executive Director, Medical Library Association, United States

## INTRODUCTION

Kevin Baliozian, CAE, MLA, is the Executive Director of the Medical Library Association (MLA), a role he has held since January 2015 when appointed by the MLA board of directors. His mandate included enhancing the association's value and relevance to its members and the broader profession and ensuring its long-term sustainability amid the challenges of the evolving health information landscape. This includes navigating challenges such as library closures, the necessity for health information professionals to develop new skills, tightening library budgets, and the impact of the COVID-19 pandemic on MLA's finances and business models.

This article examines key inflection points of the last twenty-five years and the critical role of the board of directors in setting the direction of MLA. It reviews ten years of strategic initiatives, building the larger picture of significant change for the association and the building of a better future.

## THE BOARD OF DIRECTORS COMMITMENT TO CHANGE

In December 10, 2014 twelve members of the Medical Library Association (MLA) board sat in a semicircle as I took my seat, one of two finalists for the position of executive director. This was the culmination of a months-long selection process that had started with more than 80 applicants. To prepare for the kickoff topic, “*strategic planning with a future focus, taking into consideration the generational shift*,” I had studied MLA mission statement, business plan, strategic plan, presidential priorities, and committee reports, and had spoken with several health sciences librarians for their take on the major disruptions affecting the health information ecosystem.

My twenty-minute presentation laid out the foundation of a successful strategic planning process: an MLA strategic plan should be a) consistent with MLA's mission, b) always turned to the future, and c) lead to action taking. It should identify critical high-priority areas-of-action and define several goals each with their specific objectives and metrics. An area rises to a high priority area-of-action when a) a critical set of issues require the board's attention, focus and action, b) the issues have significant and meaningful impact on MLA's ability to deliver on its mission, and c) the impact can be positive (opportunity) or negative (problem if not addressed).

I pointed to the inconsistent goals presented in the documents, the annual strategy shifts disruptive to the cohesiveness and alignment of MLA components, and the lack of a long-term focus and vision. In conclusion, I observed that 70% of the MLA strategic statements used the action word “continue.” I paused, asked the board: “*do you want to change or continue?*” and paused again.

One by one, board members expressed their commitment to changing MLA and why doing so was in their view critical. The discussion had morphed into a strategic facilitation session, as the ultimate “behavioral interview,” a technique used to assess a candidate's future performance by asking questions about past behavior in similar situations to the new roll, which would be an essential test of the alignment between the vision of the board and the fit of the executive director.

*MLA 2004–2005 board members were united in their belief and understanding that the association was at an existential inflection point, that business as usual was no option, and that there was a sense of urgency for change.*

## NAVIGATING CHANGE THROUGH DISRUPTIONS

The MLA board is elected and entrusted by its members to set the course of the association and allocate the association's limited resources to achieve those goals. *Mission* is about the relevance of MLA as an association to provide value to health information professionals, advance the health information profession as a whole, and be ahead of the curve in identifying the needs of the future. *Sustainability* is about the judicious management of the finances of the organization (revenues higher than expenses over time), and the prioritization of limited resources (volunteer time available to serve the association, staff time, and funding).

*The imperative to prioritize resources to focus on the most essential programs stems from the board and management joint responsibility to achieve both “mission” and “sustainability”. The success or failure of an association also hinges, over time, on the critical decisions in response to external disruptions.*

The following are examples of those situations from the last 25 years.

In her April 20, 1998 *Bulletin of the Medical Library Association* article *Efficiency, stability, recognition, resolution* [1], Carla J. Funk, MLS, MBA, CAE, Hon FCLIP and MLA executive director from 1992 to January 15, 2015 tells the story of MLA's transition from an all-volunteer organization to one with 18 staff members in 1996. Funk's article records the terms of MLA executive directors and describes the technology shifts affecting day-to-day operations of MLA headquarters. “When Raymond A. Palmer (1982–1991) became executive director in 1982, there was a focus on streamlining headquarters operations, strengthening dwindling financial resources, and generally doing more with less.” Financial strains were a concern during Palmer's and Funk's tenures and continue to be even 2024 as MLA staffing has decreased to 12 full-time equivalent employees while still relying heavily on volunteer members.

In their 2009 *JMLA* article *Trends in hospital librarianship and hospital library services: 1989 to 2006* Patricia L. Thibodeau, AHIP, FMLA, and Funk discussed hospital library closures [2]. They concluded that “Survey data support reported trends of consolidation of hospitals and hospital libraries and additions of new services. These services have likely required librarians to acquire new skills.” Demonstrating the value of the health information profession, especially in the clinical care setting, remains a focus of today's MLA. While it is a focus, it is nonetheless difficult to counter the continuing trend of library closures as hospital closures and consolidation have increased in the past 25 years [3].

In his 2022 MLA *Janet Doe Lecture* and subsequent *JMLA* article, *Health science libraries in the emerging digital information era: charting the course*, (Michael Kronenfeld, MLS, MBA, AHIP, FMLA, presented a retrospective and analysis of the major disruptions and resulting opportunities of digital transformation [4]. He said that “the great challenge medical library professionals are facing is how we evolve and respond to the emerging digital era. If we successfully understand and adapt to the emerging digital information environment, medical librarians/Health Information Professionals (HIPs) can play an even greater role in the advance in the health care of our nation and its residents.” That has been the case since MLA's founding on May 2, 1898, and remains the case today with the emerging and accelerating use of artificial intelligence (AI).

In his 2005 *JMLA* article *The Impact of Open Access*, T. Scott Plutchak, *JMLA* editor at the time, analyzed the effects of the 2001 decision by the MLA Board to make *JMLA* an open access journal after its content became available on *PubMed Central* [5]. On the *mission* objective, Plutchak writes: “I can think of few things more likely to gladden the heart of an editor than this kind of evidence of the reach and impact of the journal on which he lavishes so much time and attention. I have no doubt that we would not be seeing these sorts of numbers if *JMLA* were not freely available on the Web. From the standpoint of readership and reach, MLA's experiment with open access would appear to be a resounding success.”

On the *sustainability* objective, Plutchak writes, “While the loss of the *excess* revenue would not cripple the association [MLA], it would certainly require some shifting of priorities and put additional pressure on other revenue sources. If open access were to result in a significant loss of the *total* revenue, the very existence of the journal could be imperiled. The risk is not trivial.”

## A LOOMING FINANCIAL CRISIS

The financial risk to MLA identified by Plutchak did materialize. *JMLA* revenue plummeted from $526,691 (19% of total operating revenues) in 2001, to zero in 2023 when the journal fully transitioned away from print. Indeed, the MLA Board was forced to “shift priorities” as Plutchak described and utilize other revenue sources to safeguard the long-term sustainability of the journal. *JMLA* operates with an all-volunteer editorial board and today funds its costs with the revenues generated from MLA memberships and the annual conference.

By 2015, the sustainability of MLA primarily hinged on just two main revenue streams: membership dues and the annual conference. The declining number of health sciences librarians resulted in a decrease in membership, posing additional financial challenges. Specifically, *real* membership revenue fell by 28% from 2001 to 2015 when adjusting for inflation (*nominal* revenues of $642,753 in 2001 and $616,106 in 2015 adjusted by a 33% Consumer Price Index increase over the same period). Consequently, the annual conference grew in financial significance, accounting for 51% of the total operating revenue in 2015, up from 38% in 2001. This in turn intensified MLA's dependence on exhibits and sponsorships, making vendor support a critical component of MLA's financial health and introducing increased risk.

**Figure 1:**
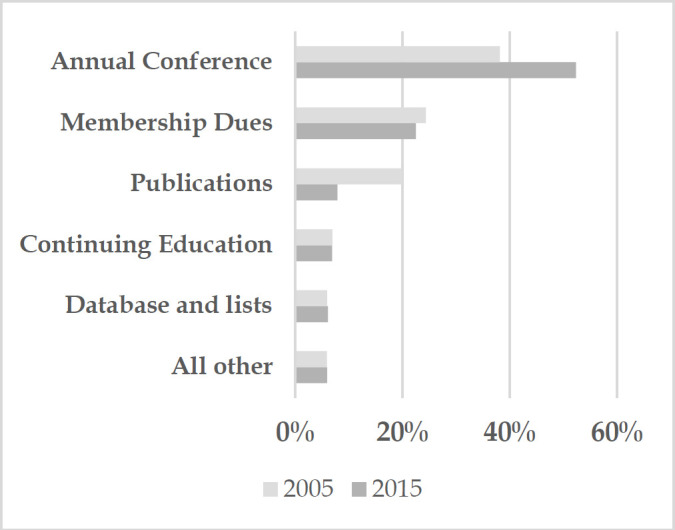
MLA Revenue Diversification 2001 vs. 2015

This graph shows MLA revenues as a percentage of total revenues. It illustrates the significant decrease in publication revenue from 2001 to 2015 offset by a corresponding increase in the annual conference, with all other revenue percentages stable. (All data from MLA 2001 and 2015 Audit Reports).

**Table 1:** MLA Revenue Diversification 2001 vs 2015 Part 2

	2001	2015
Annual Conference	$ 1,008,395	$ 1,434,087
Membership Dues	$ 642,753	$ 616,105
Publications	$ 526,691	$ 215,316
Continuing Education	$ 184,453	$ 189,185
Database and Lists	$ 157,290	$ 165,724
All other	$ 155,289	$ 163,709
Total Operating Revenue	$ 2,644,300	$ 2,738,450

**Table 2:** MLA Revenue Diversification 2001 vs 2015 Part 3

	2001	2015
Annual Conference	38%	52%
Membership Dues	24%	22%
Publications	20%	8%
Continuing Education	7%	7%
All Other	6%	6%
Database and Lists	6%	6%
Total Operating Revenue	100%	100%

By 2015, MLA's lack of diversification in its revenue streams, compounded by a trend of declining revenues due to shrinking library budgets, led to the emergence of systemic operational deficits. While financial revenues from MLA's reserves could temporarily offset these operational deficits, there was no long-term strategy in place to address the underlying issues with the MLA business model. A sustainable solution to diversify and stabilize MLA's financial sources was urgently needed to address these challenges.

The 2014–2015 board of directors opted for a *growth* strategy to achieve long-term sustainability, with a prudent use of MLA reserves to invest in the future.

*MLA had the financial reserves to transform itself and invest in its future, and it would do so by increasing the mission value AND revenues through diversification.*

A *cost-cutting* strategy would likely have resulted in an association *death spiral*: budget cuts lead to fewer MLA programs, which leads to loss of value offered by MLA, which leads to loss of engagement, which leads to loss of revenue, which leads to more program cuts.

The following graph compares the change of operating revenues, from the base year of 2005 to 2005, for MLA, the Special Libraries Association (SLA), the American Library Association (ALA) and the American Association of Law Libraries (AALL) [6].

**Figure 2:**
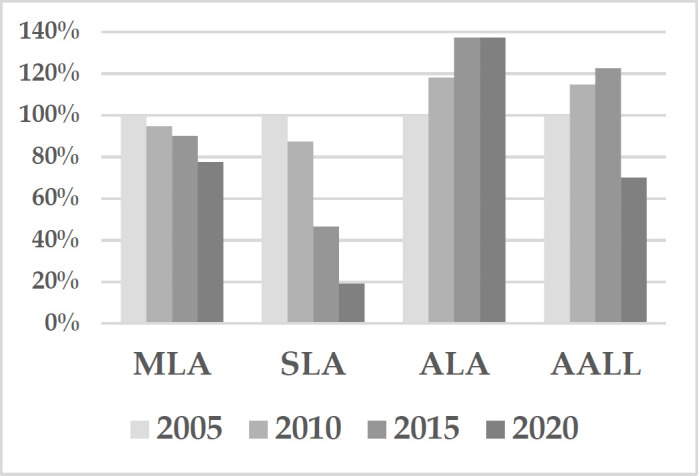
2005–2020 Operating Revenues

**Table 3:** 2005–2020 Operating Revenues Part 2

	MLA	SLA	ALA	AALL
2005	$ 3,173,308	$ 6,889,403	$ 36,435,188	$ 3,042,633
2010	$ 3,006,283	$ 6,012,166	$ 43,002,503	$ 3,491,219
2015	$ 2,856,768	$ 3,199,898	$ 49,989,270	$ 3,726,183
2020*	$ 2,459,909	$ 1,319,529	$ 50,033,474	$ 2,131,711

While MLA operating revenues decreased by 20% from 2005 to 2020, SLA revenues show a downward spiral, ALA revenues are resilient, and AALL revenues grew until Covid-19 caused the cancellation of their 2020 in-person annual conference. MLA mitigated the negative financial effects of Covid-19 on its 2020 revenues by transitioning to a successful virtual conference and by launching the all-access *passport* to MLA online education. In 2023, MLA pre-financial audit operating revenues are back above $3M [6]. (Data from public 990 filings: total revenues less financial revenue).

## SETTING THE STRATEGY

The 2014–2015 and subsequent boards of directors leveraged the analysis of the *Future's Taskforce* (2012–2014). In their October 2014 report, the taskforce recommended to a) establish areas of practice of the association, b) expand the membership base, c) transition to a year-round model less dependent on in-person annual conference, d) streamline the organizational structure (simplify, clarify, eliminate, reduce), and d) establish new positions for MLA governance such as the *Innovator-in-Residence*, the *Data Curator/Analyst* and the *Instructional Designer/Learning Technologies Coordinator*.

The transition to a year-round model less dependent on the conference meant developing a continuing education program where MLA would “take responsibility for creating courses and providing resources for members to create courses, rather than mostly approving courses created by individuals and other organizations” and would “increase time devoted to networking and programming and decrease time devoted to business meetings and other administrative functions”.

In streamlining the organization structure, the taskforce recommended to “clarify the purpose of sections and focus their activities on content rather than administration. Sections could function as *Working Groups* focused on projects that benefit the membership and profession, with an eye to creating a work product such as a webinar, program, white paper, journal article, position paper, or set of standards. Multiple sections with overlapping interests could combine talents and work on a joint project.” The taskforce also recommended to “possibly rename *Special Interest Groups* (SIGs) to *Caucuses* which implies more of a voice in the organization.”

The *Future's Taskforce* 2014 recommendations met with considerable pushback when presented, due to opposition and rising tensions among MLA members regarding the proposal to streamline Sections and SIGs. Many Section members felt that this suggestion amounted to a loss, as no alternative clear, collective vision for an improved future state of MLA communities had been convincingly presented.

*The transformation of MLA communities had started on the wrong foot in 2014, and it would take several years to achieve “buy-in” from members and participants.*

## MLA STRATEGIC GOALS (2015–2024)

The board created a strategic goal specific to the transformation of *Communities - Sections and SIGs* (May 2016 to May 2020) with key premises that were to a) encourage and facilitate community activities throughout the year, rather than just focus on programming at the annual conference, b) combine the dual structure of paid sections and open SIGs into a single set of caucuses all members could join at no extra charge, c) reintegrate the funds of individual Sections into MLA general funds, and c) sustain the funding of Section awards and scholarships by having those supported by the MLA endowment.

The board convened a diverse coalition of members to communicate, gather feedback, and refine strategies through two years of dialogue. The implementation of the *MLA Diversity, Equity, and Inclusion* (DEI) strategic goal (May 2017 to May 2020) played a crucial role in making a compelling case for community transformation.

*By eliminating the dual hierarchy between Sections and SIGs and the cost barrier to community participation (no additional fee to membership), and by merging Section treasuries to benefit the whole, the community transformation roadmap presented a strong argument for equity and inclusion, which in turn would enhance diversity within the organization.*

The objective of the *What MLA Does* strategic goal (February 2015 to May 2017) was to identify MLA programs that were strategic and relevant to members and improve or eliminate those that were not. An immediate focus was to speed up decision making and execution, and ensure that the association provided value, and did so in a financially sustainable way. An essential outcome was to define the overall vision to spawn new strategic goals aimed at improving the MLA member experience (e.g. technology, communities, diversity, equity and inclusion, annual conference) and increasing the value of MLA to the broader audience (education, areas of expertise, new audiences).

The objective of the *MLA Technology* strategic goal (February 2015 to May 2018) was to improve the online user experience and access to information by members, customers, and the public. This included growing community interactions that were, at the time, occurring across multiple and disparate websites and communication channels, many outside of the association's operations. MLA introduced new technology that centralized the association activities into a coherent experience and branding, and at a lower cost.

In June 2024, MLA introduced a new technology platform to once again significantly increase user experience and staff productivity, addressing both *mission* and *sustainability*.

The *Communities (Sections and SIGs)* strategic goal (May 2016 to May 2020) discussed above was followed by a second *Communities (part 2)* strategic goal (November 2020 to May 2023) that aimed to encourage community-driven high-quality and relevant content, ensure a professional home within MLA for all health information professionals at all career stages, empower MLA members at the grassroots level, and increase member engagement. In 2023, caucus participation by MLA members was an impressive 89%, with each participant joining an average of 4.8 caucuses [2023 MLA Business Meeting Executive Director Report].

The objective of *Annual Meeting Innovation* strategic goal (May 2018 to May 2020), extended by the *Reinvent the MLA Meeting Experience - Part 2* strategic goal (November 2020 to May 2023) was also aligned with the *Future's Taskforce* recommendations: more content, fewer association meetings, improved experience and value for broader audiences. The timing proved auspicious: the COVID-19 pandemic thrust MLA into a new era of online engagement, prompting the development of entirely virtual annual conferences in 2020 and 2021, followed by hybrid formats (in-person + live-virtual + on-demand) in subsequent years.

*The disruption brought about by the Covid-19 pandemic served as a catalyst for enduring positive changes within the association. Committees and caucuses now convene virtually throughout the year, as do MLA business meetings, the annual awards ceremony, the presidential inaugural and open forums.*

In 2018, MLA collaborated with the *National Library of Medicine* and the *Public Library Association* (a section of the *American Library Association*) to organize a 2018 symposium on *Public Health Information* as part of the MLA annual conference. This event attracted 150 public librarians with interest in public health as well as many MLA members with an interest in consumer health.

After the 2020 and 2021 hiatus of in-person annual conferences due to theCovid-19 pandemic, MLA reintroduced special content programming at the 2022 annual conference, designed for both MLA members and a broader audience, using the time slots freed up by association activities that had transitioned online. These sessions covered: *Collection Development*, *Leadership and Management*, and *Data Services and Management*.

The launch of the *Education* strategic goal (February 2015 to November 2020) was fundamental to MLA's long-term transformation, and to its resilience during the pandemic. The objectives were to position MLA as the go-to education resource for health information professionals, foster excellence in the professional practice and leadership of health sciences library and information professionals and diversify revenue outside of membership and annual conferences. Over just a few years, MLA created a structured educational curriculum with robust offerings, built on revised *MLA professional competencies* (2017) introducing measurable performance indicators by skill level, with an effective collaboration between volunteer committees, headquarters staff, subject matter experts and instructional designers. MLA's approach to education was now structured and intentional, supported by technology and marketing.

In her 2022 article *Partnering for education and career development of librarians and information specialists*, Ruth Holst, AHIP, FMLA, describes the collaboration between MLA and the *National Network of Libraries of Medicine*(NNLM) for the creation of two MLA specializations: *Consumer Health Information Specialization* (CHIS) in 2001, and the *Disaster Information Specialization* (DIS) in 2012 [6].

The development of MLA specializations is the result of MLA's rigorous process to define essential skills and performance indicators for specific areas of practice deemed central by the organization. This process includes targeted educational programs to help health information professionals acquire those skills, some in partnership with NNLM. MLA launched the *Data Services Specialization* (DIS) in 2021, and the *Systematic Review Services Specialization* (SRSS) in 2022.

The objectives of the *Research Imperative* strategic goal (May 2015 to May 2018) set a high bar for MLA excellence to a) positively impact institutional and stakeholder outcomes, such as impacts on clinical care, student learning, and scientific research, b) improve the quality of health information services through the use, creation, and application of evidence in daily practice and processes, c) foster a culture of employer support for evidence-based practice, assessment, and related research, and d) position MLA as the voice for evidence-based practice and matters related to research and statistics about health sciences libraries and librarians.

The initiative led to the establishment of the *MLA Research Training Institute* (RTI) supported by two grants from the *Institute of Museum and Library Services* (IMLS). The RTI is dedicated to fundamentally advancing health information research and promoting evidence-based practices in healthcare [8]. From 2018 to 2023, more than 100 participants have completed the year-long program, and 35 are participating in 2024.

In 2020, MLA assumed ownership of the *Electronic Fund Transfer System* (EFTS) from the *University of Connecticut Health Center* (UCHC). UCHC, in collaboration with NLM, had launched the initial version of EFTS in 1996. While NLM's *DOCLINE* system matches lenders and borrowers for interlibrary loans (ILLs), MLA's EFTS serves as the online billing system for ILLs that facilitates the financial transactions. The board's strategic decision to develop and launch a new version of EFTS was pivotal in providing support to all libraries that must manage limited subscription resources, from large academic institutions to non-academic and smaller libraries. This initiative also significantly contributed to MLA's goal of diversifying its revenue streams.

The objective of the *Building a Better Future* strategic goal (December 2020 to 2024) was to honor 125 years of MLA history and envision the future of the profession. In their JMLA article, “Welcome to the Future: Challenges and Opportunities Discussed in the Vision 2048 Task Force Open Forums 2021–2023” the *2048 Task Force* shares the results from surveys, interviews, and open comment sessions regarding the future of medical librarianship and the future of the association [9].

In launching the 2024 *Artificial Intelligence Imperative* strategic goal (May 2024 to May 2027), the board seeks for MLA to advance and promote health information practices with regards to the use of artificial intelligence (AI) by developing and implementing strategies to advance the AI skills and competencies of health sciences professionals, and advocate to employers, members, publishers, and the public the value, impact, and benefits of health sciences libraries and librarians in an AI world.

## CONCLUSION

In exercising its duty of care, the board need only be *careful* and not *right*. Nevertheless, the strategic nature of the MLA board, the thorough and informed process it applies to reach its decision, its willingness to experiment, accept failure, and adapt have all contributed to an impressive *mostly right* record over the last fifteen years.

This series of board strategic decisions has been pivotal in strengthening MLA. While further creativity and diligent effort are necessary to fully realize MLA's transformation, these efforts have yielded substantial results. The association has experienced robust growth since 2022, enhanced relevance and value to member and non-member communities, and thrives on a vibrant culture characterized by engagement, inclusivity, and trust of its members and larger health sciences community.
